# The effect of myocardial fibrosis on ventricular remodeling following valve replacement for severe aortic stenosis. A CMR study comparing transcatheter aortic valve implantation and surgical aortic valve replacement

**DOI:** 10.1186/1532-429X-14-S1-O71

**Published:** 2012-02-01

**Authors:** Timothy Fairbairn, Christopher D Steadman, Adam N Mather, Manish Motwani, Daniel Blackman, Sven Plein, Gerry P McCann, John P Greenwood

**Affiliations:** 1 Multidisciplinary cardiovascular research, University of Leeds, Leeds, UK; 2 Cardiology, Leeds General Inirmary, Leeds, UK; 3 Cardiovascular sciences, University of Leicester, Leicester, UK

## Summary

Myocardial fibrosis (MF) that occurs in aortic stenosis (AS) has been shown to be associated with a poor prognostic outcome post surgical aortic valve replacement (SAVR) but not in the high risk patients suitable for Transcatheter aortic valve implantation (TAVI). Using the technique of late-gadolinium enhancement we have shown that MF regresses post-TAVI but not post-SAVR, and that ejection fraction rather than MF is the independent predictor of reverse remodeling.

## Background

Myocardial Fibrosis (MF) occurs as part of the remodelling process in left ventricular hypertrophy associated with severe aortic stenosis (AS). MF has been shown to be associated with reduced left ventricle (LV) ejection fraction and poor prognosis following surgical aortic valve replacement (SAVR). Transcatheter aortic valve implantation (TAVI) has become a standard procedure for the treatment of severe aortic stenosis in high risk or inoperable patients. The significance of MF in this high-risk patient population has to date not been assessed. We aimed to assess the baseline burden of MF, its change following TAVI and SAVR, and its influence on LV reverse remodeling.

## Methods

Fifty high-risk patients (EuroSCORE ≥20) with severe AS (peak velocity >4m/s) underwent TAVI (n=25) or SAVR (n=25). Steady-state free procession short axis (SA) cine imaging was performed to determine LV mass and volumes. MF was assessed by late-gadolinium enhancement (LGE) in 47 patients. A look-locker sequence was performed 10 minutes following the administration of 0.2mmol/kg of Gadoteric acid (Doteram, Guerbet, SA, Villepinte). The inversion time was individually adjusted and 10-12 short axis slices (10mm thickness, matrix 240x240, typical FOV 340mm) were acquired.

## Results

**Figure 1:**
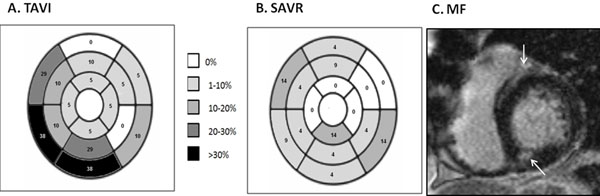
Myocardial fibrosis burden (%) of the TAVI (A) and SAVR (B) patients, as represented on a segmental level according to the American Heart Association 16 segment models. An example of the fibrosis in AS on late gadolinium enhancement is shown (C, white arrows).

MF was detected in 25 (53%) patients (TAVI 13(59%) vs. SAVR 12(48%), P=0.38). Fibrosis was predominantly found in the basal region and septal segments for both groups. MF burden was greater in the TAVI group (10% (35/352) of segments) compared to the SAVR group (5% (19/400) segments), (P=0.01), Figure 1. Hypertension was associated with an increased risk of baseline MF by logistic regression (OR 5.9, P=0.015). Baseline MF burden was positively associated with higher end-diastolic (B 5.4, P<0.001), end-systolic (B 6.7, P<0.001) volumes, greater mass (B 5.6, P=0.002) and negatively associated with ejection fraction (B -3.9, P<0.001). MF predicted a greater decrease in ESV (B -7.6, P<0.001) and increase EF (B 1.8, P=0.008) post-TAVI and SAVR by univariate analysis. These became insignificant on multivariate analysis as baseline EF was the dominant predictor of improved EF (B-0.49, P<0.001). Fibrosis regressed post-TAVI (35(10%) vs. 21(6%) segments, P=0.04) but not post-SAVR (19(5%) vs. 16(4%) segments, P=0.32). TAVI (OR 3.8, P=0.039) was associated with an increased likelihood of post-operative MF regression.

## Conclusions

MF burden is associated with poor baseline LV volumes and function. Baseline EF rather than MF independently predicts reverse remodeling post TAVI and SAVR. Significant regression of fibrosis occurred only post-TAVI. This reduction of fibrosis may be important in improving prognosis following aortic valve replacement.

## Funding

None.

